# A protocol for determining cage-escape yields using nanosecond transient absorption spectroscopy

**DOI:** 10.1016/j.xpro.2023.102312

**Published:** 2023-05-13

**Authors:** Alexia Ripak, Simon De Kreijger, Benjamin Elias, Ludovic Troian-Gautier

**Affiliations:** 1 Université catholique de Louvain (UCLouvain). Institut de la Matière Condensée et des Nanosciences (IMCN), Molecular Chemistry, Materials and Catalysis (MOST), Place Louis Pasteur 1, bte L4.01.02, 1348 Louvain-la-Neuve, Belgium

**Keywords:** Energy, Chemistry, Material Sciences

## Abstract

Here, we present a protocol for the determination of cage-escape yields following excited-state electron transfer between a photosensitizer and a quencher. We describe steps for determining changes in molar absorption coefficient of the different oxidation states via photolysis experiments and the percentage of reacted species via steady-state or time-resolved spectroscopy. We then detail measurement of the amount of formed product via nanosecond transient absorption spectroscopy.

For complete details on the use and execution of this protocol, please refer to Ripak et al. (2023).^1^

## Highlights

• A protocol to determine cage-escape yields in solution is described
• Protocol is compatible with oxidative or reductive excited-state electron transfer
• Two approaches used depending on the photosensitizer’s and quencher’s stability
• Photolysis as alternative to spectroelectrochemistry and chemical redox reactions

## Abstract (editor highlights)

Publisher’s note: Undertaking any experimental protocol requires adherence to local institutional guidelines for laboratory safety and ethics.

## Graphical abstract

**Figure:**
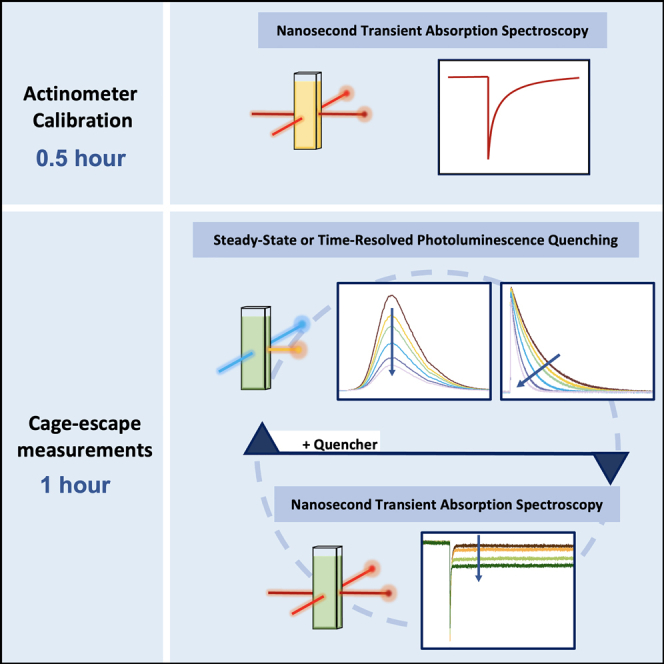

## Before you begin

The protocol describes the specific steps to determine cage-escape yields following excited-state electron transfer between [Ru(bpy)_3_]^2+^ (the photosensitizer, PS) and 4-methoxy-benzene diazonium (the quencher, Q), using [Ru(bpy)_3_]^2+^ as actinometer, i.e., a species used to gauge the radiation intensity. The experiments were performed in acetonitrile, but any other inert, non-absorptive solvent can be used as long as they allow a complete solubility of the photosensitizers and quenchers. Note that, although described for oxidative electron transfer, this method was also used to determined cage-escape yields of reactions proceeding via reductive excited-state electron transfer.^1^^,^^2^^,^^3^^,^^4^^,^^5^^,^^6^^,^^7^^,^^8^^,^^9^^,^^10^^,^^11^^,^^12^^,^^13^^,^^14^^,^^15^^,^^16^^,^^17^^,^^18^^,^^19^^,^^20^^,^^21^^,^^22^^,^^23^^,^^24^^,^^25^^,^^26^^,^^27^^,^^28^^,^^29^^,^^30^^,^^31^^,^^32^^,^^33^^,^^34^^,^^35^ We describe the sequential steps that are needed to perform these cage-escape yields measurements. The first part consists in determining changes in molar absorption coefficient of the photosensitizer in different oxidation states using photolysis. The second step describes how to determine the percentage of quenched photoluminescence and how to measure the concentration of products formed after excited-state electron transfer. Two methods for the determination of cage-escape yields are reported. One method uses one cuvette of PS with increased concentrations of quencher while the other method uses individual cuvette for each concentration of quencher.

### Photolysis experiment

**Timing: 0.5–1 h**

Photolysis experiments are performed to determine Δε_PS+_, i.e., the difference in molar absorption coefficient between the PS in its ground and oxidized states (ε_PS+_ – ε_PS_). This value is required to determine cage-escape yields and will be further discussed in the quantification and statistical analysis section (*vide infra*). The light-induced reaction proceeds according to the following equations:

(Equation 1) $${{[{Ru}^{II}\left(bpy\right)}_{3}]}^{2+}\;\overset{h\nu }{\to }{\left[{Ru}^{III}{\left(bpy\right)}_{2}\left({bpy}^{\cdot –}\right)\right]}^{2+*}\left(Excitation\right)$$

(Equation 2) $${\left[{Ru}^{III}{\left(bpy\right)}_{2}\left({bpy}^{\cdot –}\right)\right]}^{2+*}+{R-N}_{2}^{+}\;\overset{et}{\to }{{[{Ru}^{III}\left(bpy\right)}_{3}]}^{3+}+{R-N}_{2}^{\cdot }\;\left(Electron\;transfer\right)$$

(Equation 3) $${{[{Ru}^{III}\left(bpy\right)}_{3}]}^{3+}+{R-N}_{2}^{\cdot }\to {{[{Ru}^{III}\left(bpy\right)}_{3}]}^{3+}+R^{\cdot }+N_{2}\;\left(Dissociation\right)$$

(Equation 4) $${{[{Ru}^{III}\left(bpy\right)}_{3}]}^{3+}+R^{\cdot }\to {{[{Ru}^{III}\left(bpy\right)}_{3}]}^{3+}+R\;\left(Termination\right)$$

**Figure 1:**
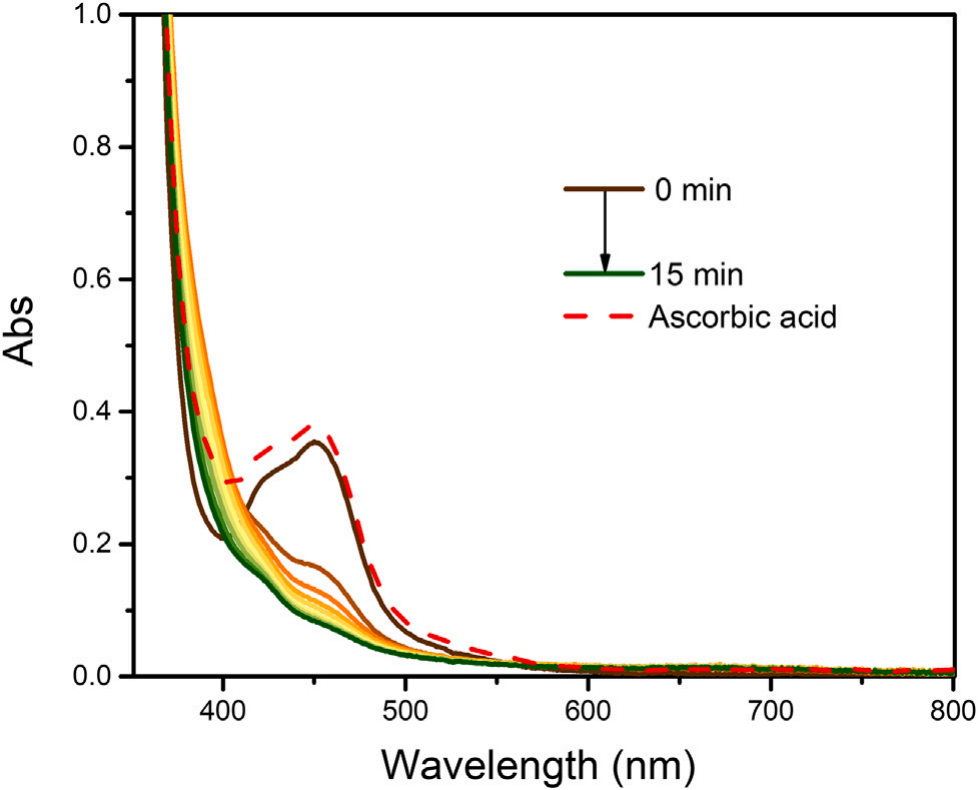
Photolysis of [Ru(bpy)_3_]^2+^ in the presence of 4-methoxy-benzene diazonium Experiments were carried out is argon purged acetonitrile with 30 mM of 4-methoxy-benzene diazonium. The dashed curve corresponds to the spectrum recorded after the addition of L-ascorbic acid. The discrepancy between the original spectra and the one after addition of L-ascorbic acid originates from the absorption tail of excess 4-methoxy-benzene diazonium.

Light absorption populates a triplet metal-to-ligand charge transfer (^3^MLCT) excited state (Equation 1) that undergoes oxidative electron transfer (Equation 2) to form the corresponding oxidized [Ru(bpy)_3_]^3+^ and reduced aryl-diazonium derivative. This radical then dissociates to generate nitrogen and the corresponding aryl radical (Equation 3) that reacts in an undefined manner in the present case (Equation 4) leading to the accumulation of [Ru(bpy)_3_]^3+^. UV-visible spectra of a PS solution (20–60 μM) with an excess (at least 100 times more concentrated) of sacrificial quencher are recorded at selected time intervals following irradiation until absorption changes are no longer observed (Figure 1). Conversion of absorption spectra into molar absorption coefficient via the Beer-Lambert Law allows us to calculate the corresponding Δε spectra and determine the value of Δε for any wavelength of interest within the absorption limit of the Beer-Lambert law. Similar experiments can be performed for reductive electron transfer using electron donors such as EDTA, triethylamine or BIH for example.^36^ Note however that reversibility of the reaction must also be assessed and confirmed to obtain rigorous results (*vide infra*).

1. Prepare a 3 mL solution of the photosensitizer (PS) in acetonitrile with an absorbance between 0.3 and 0.6 at λ_max_ in the visible region. This corresponds to a concentration range of PS between 20 and 60 μM.
2. Place the solution in a (quartz) cuvette with four optical faces and seal it with a septum and parafilm.
  a. Purge the solution with argon for 10 min and record an absorption spectrum in the appropriate wavelength range.
  b. Open the cuvette, quickly add 22mg of 4-methoxy-benzene-diazonium (30 mM).
  c. Reseal the cuvette with a septum.
  d. Parafilm and purge the solution with argon for 2 min.
  e. Record another absorption spectrum before starting the photolysis.
3. Irradiate the cuvette with a LED strip or LED lamp at a wavelength close to the photosensitizer’s λ_max_ in the visible region.

***Note:*** For [Ru(bpy)_3_]^2+^, λ_max_ = 450 nm and is hence illuminated with blue LED.

4. Record a UV-Vis absorption spectrum at selected time intervals until no further changes are observed. At this point, the absorption spectrum of the mono-oxidized PS is obtained.

***Optional:*** After complete oxidation, remove the septum and add an excess of electron-acceptor, L-ascorbic acid in this case, to assess the redox process reversibility. If the PS is stable in its oxidized form, and that the electron donor does not absorb it that range, the original spectrum should be recovered. If reductive electron transfer was investigated, an electron acceptor needs to be added, such as K_2_S_2_O_8_, for example.

***Note:*** All the L-ascorbic acid might not be soluble in acetonitrile. It is important to let the solid settle before recording an absorption spectrum to avoid any scattering.

***Note:*** The cuvettes used in the present study have a 24/40 joint that can be easily sealed with a septum, Teflon tape and parafilm.

***Note:*** Purging is performed using an argon tank connected to a bubbler filled with acetonitrile and is then connected to the cuvette. This allows to control the gas flow as well as avoid changes in concertation due to evaporation. Nitrogen can also be used instead of argon following the same procedure and controls.

***Note:*** Δε_PS+_ are more adequately determined by spectroelectrochemistry,^37^^,^^38^^,^^39^ but this equipment is not always available in the laboratory. The photolysis approach presented here is an affordable and reliable alternative to determine Δε_PS+_. Alternative methods using chemical oxidant are also possible.^39^^,^^40^

### Determining the optimal operating wavelengths

**Timing: 0.5 h**

Cage-escape experiments require multiple spectroscopic measurements at different wavelengths. λ_exc_ is the excitation wavelength that must be common for both the actinometer and the sample. λ_det_ is the wavelength at which the transient absorption signals will be detected. λ_det_ can vary for each photosensitizer and can be different from the actinometer.

5. Determine λ_exc_:
  a. Record a UV-visible absorption spectrum of the actinometer and the PS.
  b. Determine λ_exc_: it should be a wavelength where both the actinometer and the PS absorb reasonably well.

**Figure 2:**
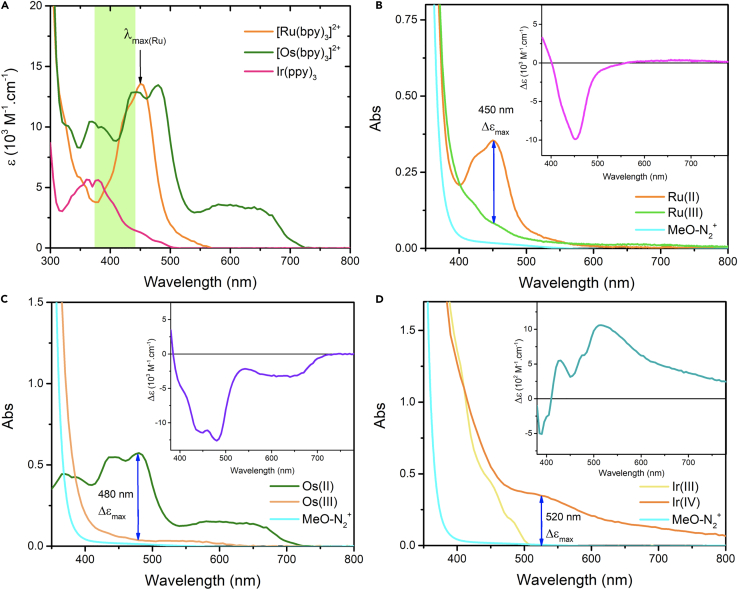
UV-Visible absorption spectra of relevant photosensitizers and quenchers (A) In green, illustration of the possible excitation wavelengths for [Ru(bpy)_3_]^2+^, [Os(bpy)_3_]^2+^ and [Ir(ppy)_3_] in acetonitrile. (B–D) Determination of λ_det_ for (B) [Ru(bpy)_3_]^2+^, (C) [Os(bpy)_3_]^2+^ and (D) [Ir(ppy)_3_] using molar extinction coefficients difference, also illustrated in the corresponding insets. Experiments were carried out in argon-purged acetonitrile.

**CRITICAL:** λ_exc_ should be different from 450 nm that is used as the λ_det_ for the actinometer in the present case.

***Note:*** In the example in Figure 2A, the highlighted green zone represents the wavelengths that can be used to excite [Ru(bpy)_3_]^2+^ and two others PS. For example, a good irradiation wavelength for [Ru(bpy)_3_]^2+^, [Os(bpy)_3_]^2+^ and [Ir(ppy)_3_] is λ_exc_ = 420 nm.

6. Determine λ_det_:
  a. Superimpose the data for the photosensitizer and its oxidized form as determined through the photolysis experiment.
  b. A good value for λ_det_ is where the ΔAbs, and thus the Δε, of the PS and PS^+^ is maximized (Figures 2B–2D).

***Note:*** For [Ru(bpy)_3_]^2+^, a good λ_det_ is 450 nm. This wavelength is used for the actinometer calibration but also for [Ru(bpy)_3_]^2+^ as a photosensitizer. For [Os(bpy)_3_]^2+^ and [Ir(ppy)_3_], λ_det_ of 480 and 520 nm can be used safely to give good transient absorption measurements, respectively.

## Key resources table

**Table:** 

REAGENT or RESOURCE	SOURCE	IDENTIFIER
**Chemicals, peptides, and recombinant proteins**		

4-Methoxybenzenediazonium tetrafluoroborate 98%	Thermo Scientific Chemicals	Cat#15422188; CAS: 459-64-3
Acetonitrile 99.9%	VWR	Cat#83639.320; CAS: 75-05-8
[Ru(bpy)_3_]^2+^.2PF_6_	B. Elias synthesized according to Ref.^41^	CAS: 60804-74-1
Argon	Alphagaz	Cat#P0021

**Software and algorithms**		

Microsoft Excel	www.microsoft.com	N/A
OriginPro	www.originlab.com	N/A

**Other**		

Blue LED, 470nm, 4.0 mW/cm^2^	Thorlabs	Cat#LIU470A
1mL Gastight Syringe Model 1001 TLL, PTFE Luer Lock	Hamilton	Cat#81320
Nanosecond transient absorption	Edinburgh Instruments	Cat#LP980-K
Spectrophotometric Quartz Cuvette with 24/40 Jt.	Quark Glass	Cat#QSE-5-4
Fluorimeter Cary Eclipse	Varian	N/A

## Materials and equipment

Nanosecond transient absorption measurements were recorded on a LP980-K spectrometer from Edinburgh Instruments equipped with an iCCD detector from Andor (DH320T). The excitation source was a tunable Nd:YAG Laser NT342 Series from EKSPLA. The third harmonic (355 nm) at 150 mJ was directed into an optical parametric oscillator (OPO) to enable wavelength tuning starting from 410 nm. The laser power was then attenuated to reach appreciable signal/noise and the integrity of the samples was verified by UV–Vis measurements. The LP980-K is equipped with a symmetrical Czerny-Turner monochromator. For single wavelength absorption changes, a 1800 g mm^–1^ grating, blazed at 500 nm is used, which affords wavelength coverage from 200 to 900 nm. For spectral mode (iCCD), a 150 g mm^–1^ grating, blazed at 500 nm is used, offering a wavelength coverage of 540 nm over the full wavelength range extending from 250 to 900 nm. Single wavelength absorption changes were monitored using a PMT LP detector (Hamamatsu R928) which covers the spectral range from 185 to 870 nm. The probe was a 150 W ozone-free xenon short arc lamp (OSRAM XBO 150W/CR OFR) that was pulsed at the same frequency as the laser. An average of 30 scans per measurement was used.

## Step-by-step method details

### Sample preparations

**Timing: 0.5-1 h**

1. Prepare a stock solution of the photosensitizer (PS) in acetonitrile with an absorbance between 0.3 and 0.6 at λ_exc_.

***Note:*** A stock solution of 6 mL is needed for experiments requiring only one cuvette for all data points, whereas 20 mL are needed to obtain 5 data points with individual cuvettes.

2. Prepare 3 mL of the reference actinometer.

***Note:*** the prototypical [Ru(bpy)_3_]^2+^ is usually used for cage-escape yields determination with absorbance values between 0.3 and 0.6 at λ_exc_ in acetonitrile.

3. Weight the quencher to prepare 20 mM solution in 2 or 4 mL of the stock solution of the PS in acetonitrile (see method 1 and 2 below).

***Note:*** The quencher solution is prepared just before the beginning of the experiment. If the quencher is not used immediately after weighing, make sure to keep it under stable storing conditions (refrigerated (at 4°C) in dark conditions for this example).

***Note:*** The quencher solution is prepared in the PS stock solution to prevent changes in absorption due to dilution during the titration.

### Actinometer calibration

**Timing: 0.5 h**

The actinometer, a solution of [Ru(bpy)_3_]^2+^ in this case, is used as external reference for the cage-escape measurements. Calibration of the actinometer with the nanosecond transient absorption spectrometer allows a comparative analysis between different pairs of photosensitizers/quenchers and the actinometer. Once set up, λ_exc_ and laser power intensity must remain unchanged for all the transient absorption measurements.

4. Place 3 mL of the [Ru(bpy)_3_]^2+^ stock solution in a (quartz) cuvette with four optical faces.
  a. Seal the cuvette with Teflon tape, a septum and parafilm.
  b. Purge the cuvette with argon for 10 min.
  c. Record the absorbance value at λ_exc_.

**CRITICAL:** Efficient Argon-purging of the analyzed solution is a key parameter to control errors on the experiments. Measuring the actinometer excited-state lifetime as a function of purging time until it remains unaltered will help to find the minimum time needed to efficiently purge the solution.

5. Calibrate the laser power by nanosecond transient absorption measurement.

**Figure 3:**
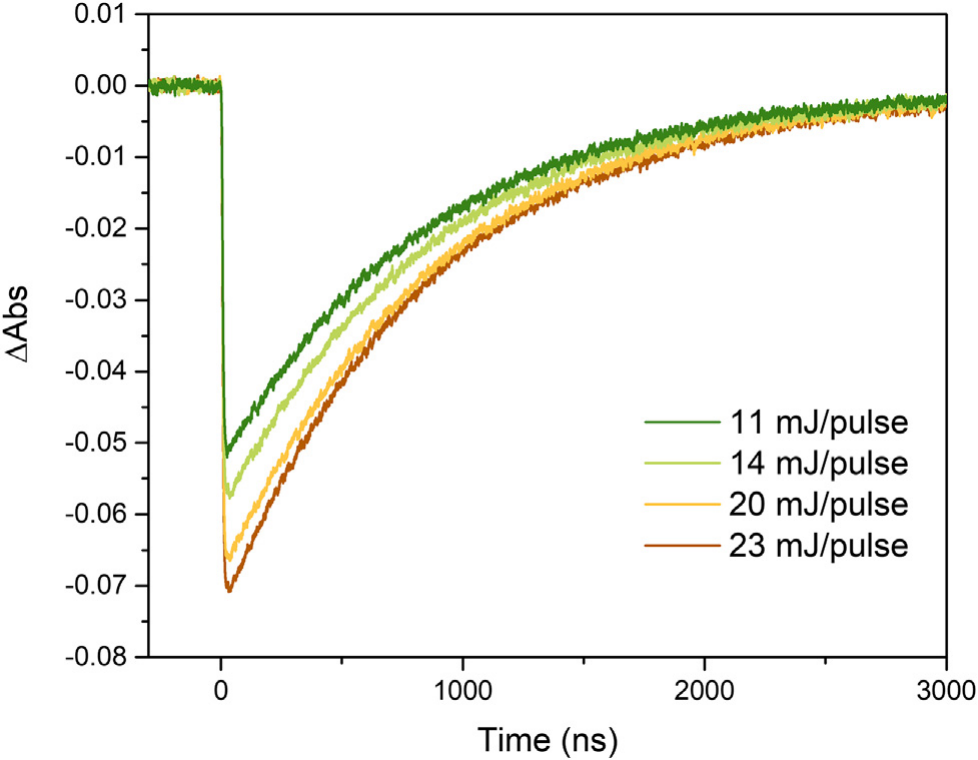
Impact of laser power on single wavelength absorption changes Experiments were carried out using [Ru(bpy)_3_]^2+^ in acetonitrile. λ_exc_ = 420 nm; λ_det_ = 450nm. The laser pulse diameter was 5 mm.

***Note:*** Calibration is performed by using different laser intensities in order to define the optimal laser power (Figure 3). Here, a 60% laser intensity was used, corresponding to a laser power of 14 mJ/pulse.

***Note:*** Saturation can appear with less than 100% laser power. ΔAbs signal should increase linearly with laser power intensity. If the intensity of the signal remains unaltered upon increasing laser power, saturation of the excited state has been reached and a less intense laser power should be used. This parameter depends on the laser itself. It is preferable to work with a moderate laser power to ensure the correct calibration of the actinometer.

6. Record the single-wavelength absorption changes of the actinometer using the previously determined laser power as well as λ_exc_ and λ_det_.

***Note:*** It is recommended to repeat step 6 at regular intervals during the experiment to ensure constant laser power irradiation.

### Cage-escape measurements

**Timing: 1 h per experiment**

Two methods can be used to record cage-escape yields using a combination of steady-state or time-resolved photoluminescence and nanosecond transient absorption spectroscopy. The choice between the two methods depends on the relative stability of the photosensitizer and quencher in the dark and under irradiation. The first method consists in preparing one spectrophotometric cuvette with the photosensitizer and adding multiple aliquots of quencher solution. The second method consists in preparing a series of spectrophotometric cuvettes with an identical concentration of photosensitizer and different concentrations of quencher.

**Figure 4:**
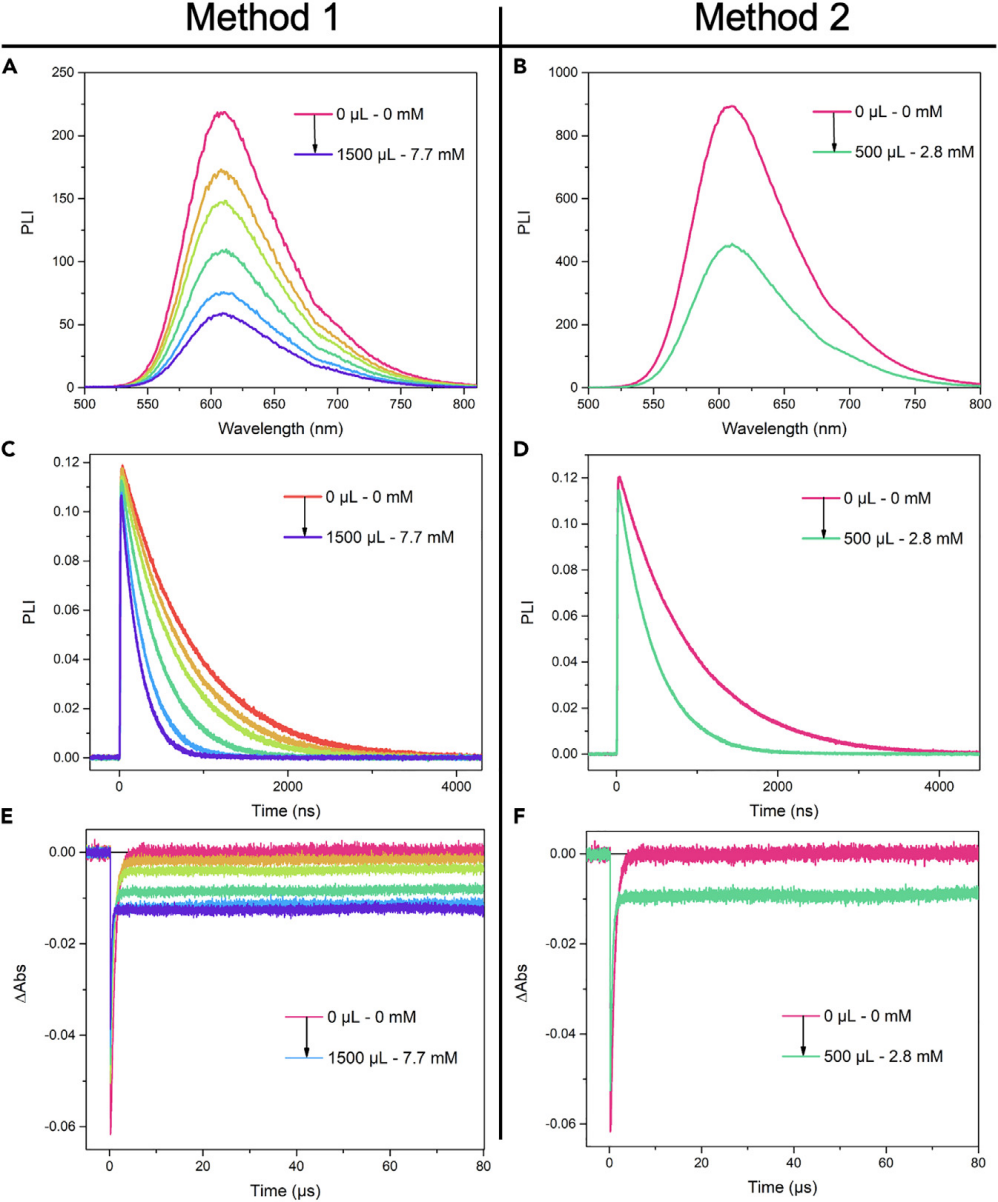
Quantification of excited-state reactivity between [Ru(bpy)_3_]^2+^ and 4-methoxy-benzene diazonium tetrafluoroborate (A, B) Steady-state photoluminescence spectra, (C, D) time-resolved luminescence quenching experiments of [Ru(bpy)_3_]^2+^ with increasing concentration (A, C) or with 2.8 mM (B, D) of 4-methoxy-benzene diazonium tetrafluoroborate. (E and F) Single wavelength transient absorption changes of [Ru(bpy)_3_]^2+^ recorded at 450 nm following pulse light excitation at the same concentrations in quencher as in panel A–D. Experiments were recorded under argon in acetonitrile at room temperature.

7. Method 1.
  a. Introduce 3 mL of the PS stock solution in the (quartz) cuvette with four optical faces.
  b. Seal the cuvette with Teflon tape, a septum and parafilm.
  c. Purge the solution with argon for the required amount of time.
  d. Record the absorbance at λ_exc_.
  e. Record a steady-state photoluminescence spectrum (a complementary excited-state lifetime decay can also be recorded if desired).
  f. Dissolve the previously weighted quencher in 2 mL of the PS stock solution and seal the vial with a septum and parafilm.
  g. Purge the solution with argon for the required amount of time.
  h. Add consecutive aliquots of quencher solution into the spectrophotometric cuvette using a Hamilton syringe to reach total added volumes of 100, 200, 500, 1000 and 1500 μL.
  i. For each addition:
    i. Record the absorbance at the excitation wavelength (λ_exc_).
    ii. Record the steady-state photoluminescence spectrum.
    iii. Record the excited-state lifetime decay.
    iv. Record the single wavelength (λ_det_) transient absorption changes (Figures 4A, C and E).
      ***Note:*** It is important to work in a concentration range that allows significant variations of the quenching percentage and ΔAbs with increasing quencher concentration.
      ***Note:*** The timescale used to record the single wavelength transient absorption changes is increased compared to the one used for the actinometer. In this case, a timescale of 100 μs was used.
      **CRITICAL:** The cuvette orientation was also controlled in the different devices. One face of the cuvette was marked and was always oriented in the same direction in the sample holder of the different devices.

8. Method 2.
  a. Prepare five (quartz) cuvettes with four optical faces, each one containing 3 mL of the stock PS solution.
  b. Seal the cuvette with Teflon tape, a septum and parafilm.
  c. Purge the solutions with argon for the required amount of time.
  d. Dissolve the previously weighted quencher in 4 mL of the PS stock solution and seal the vial with a septum and parafilm.
  e. Purge the solution with argon for the required amount of time.
  f. Record the absorbance at λ_exc_ of the cuvette without quencher.
  g. Record a steady-state photoluminescence spectrum (a complementary excited-state lifetime decay can also be recorded if desired) of the cuvette without quencher.
  h. Add 100 μL of the quencher stock solution.
  i. Record the absorbance at λ_exc_ of the spectrophotometric cuvette with the quencher.
  j. Record a steady-state photoluminescence spectrum (a complementary excited-state lifetime decay can also be recorded if desired) of the spectrophotometric cuvette with the quencher (Figures 4B and 4D).
  k. Record the single wavelength (λ_det_) transient absorption changes (Figure 4F).
  l. Repeat steps c-h with the other cuvettes but change the added volume of quencher stock solution (step h) to 200, 500, 1000 and 1500 μL respectively.

***Note:*** It is important to work in a concentration range that allows significant variations of the quenching percentage and ΔAbs with increasing quencher concentration.

***Note:*** The timescale used to record the single wavelength transient absorption changes is increased compared to the one used for the actinometer. In this case, a timescale of 100 μs was used.

**CRITICAL:** Sealing tightly the spectrophotometric cuvettes is of paramount importance for these measurements as oxygen can impact the amount of excited-state quenching, product formation and recombination processes after electron transfer. It is advisable to use Teflon tape, a new septum and parafilm to seal the cuvette for each measurement.

**CRITICAL:** The cuvette orientation was also controlled in the different devices. One face of the cuvette was marked and was always oriented in the same direction in the sample holder of the different devices.

***Note:*** Method 1 is less time-consuming and more convenient when no stability issue arises (see troubleshooting). Method 2 is more consuming in terms of materials and time but allows to limit problems due to stability issues.

## Expected outcomes

This protocol allows us to determine the cage-escape yields, i.e., the efficiency with which the formed geminate radicals pair separate and escape the solvent cage after excited-state electron transfer. With the values obtained herein, the operators should be able to determine cage-escape yields around 35% for the light-activated electron transfer reaction between [Ru(bpy)_3_]^2+^ and 4-methoxy-benzene diazonium tetrafluoroborate.

## Quantification and statistical analysis

In here, we describe how the above-mentioned protocols and corresponding collected data are used to determine the cage-escape yields.

1. Determine the relative yield of PS^+^ formed (Ф) at each concentration of quencher (Equation 5).

The maximum absorption changes generated from the oxidized PS (${\Delta A}_{PS^{+}}$) was compared to the absorption maxima of the excited state of the reference [Ru(bpy)_3_]^2+^ (${\Delta A}_{ES_{ref}}$) and normalized by their respective absorptances at the excitation wavelength (λ_exc_). A Δε value of –11000 M^–1^cm^–1^ for the actinometer at 450 nm was used.^40^^,^^42^^,^^43^ Δε_450nm_ = –9000 M^–1^cm^–1^ for [Ru(bpy)_3_]^3+/2+^ was determined through the photolysis experiments. The different values used in this protocol are gathered in Table 1.

(Equation 5) $$\Phi =\left(\frac{\frac{{\Delta A}_{PS^{+}}}{{\Delta \varepsilon }_{PS^{+}}}}{\frac{{\Delta A}_{ES_{ref}}}{{\Delta \varepsilon }_{ES_{ref}}}}\right)\left(\frac{1-10^{-Abs_{ref}\left(\lambda_{exc}\right)}}{1-10^{-Abs_{PS}\left(\lambda_{exc}\right)}}\right)$$

2. Plot the values of Ф against %PL Quenched (Equation 6).

**Table 1:** Representative data obtained for one cage-escape experiment using [Ru(bpy)_3_]^2+^ and 4-methoxy-benzene diazonium tetrafluoroborate

V_Q_ added (μL)	%PL Quenched^a^	${\Delta A}_{PS^{+}}$ (x10^-3^)	${\Delta \varepsilon }_{PS^{+}}$ at λ_det_	${\Delta A}_{ES_{ref}}$	${\Delta \varepsilon }_{ES_{ref}}$ at 450 nm	$Abs_{ref}$ at λ_exc_	$Abs_{PS}$ at λ_exc_	Ф
0	0	0	–9000	–0.055	–11000	0.231	0.241	0
100	10	–0.7	–9000	–0.055	–11000	0.231	0.246	0.02
200	24	–3.29	–9000	–0.055	–11000	0.231	0.247	0.07
500	47	–7.87	–9000	–0.055	–11000	0.231	0.253	0.16
1000	66	–11.4	–9000	–0.055	–11000	0.231	0.267	0.23
1500	74	–11.6	–9000	–0.055	–11000	0.231	0.264	0.23

a Determined using the integrated photoluminescence spectra or the excited-state lifetime (see below).

**Figure 5:**
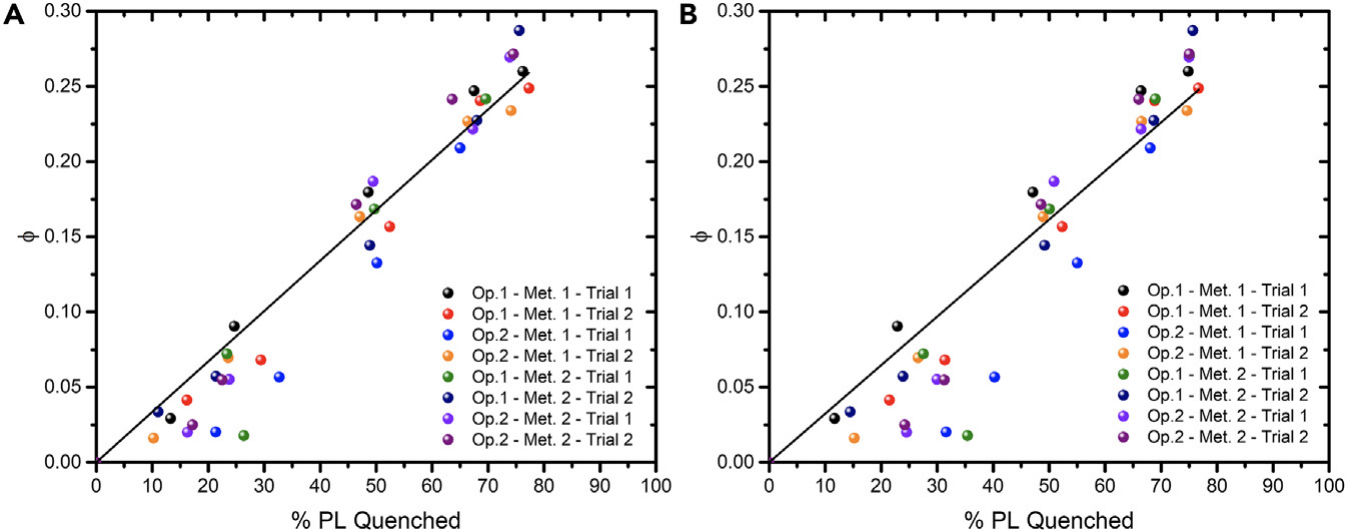
Determination of cage-escape yields for the excited-state reaction between [Ru(bpy)_3_]^2+^ and 4-methoxy-benzene diazonium tetrafluoroborate (A and B) Plots of the relative yield of PS^+^ formed (φ) versus the percentage of quenched steady-state (A) and time-resolved (B) photoluminescence (% PL quenched) for [Ru(bpy)_3_]^2+^ in the presence of 4-methoxy-benzene diazonium tetrafluoroborate. Experiments are presented for operators 1 and 2 (Op.) according to methods 1 and 2 (Met) and the indicated trial. The slope was used to extract a cage-escape yield of 34% (a) and 32% (b). An error of ca. 10% is estimated based on uncertainties associated with the changes in molar absorption coefficient of the actinometer and the oxidized photosensitizers as well as with the trial averages. An operator unfamiliar with these experiments also obtained φ_CE_ of 34% and 32% using method 1 and 2 of this protocol, respectively.

Final cage-escape yields (φ_CE_) values are obtained by comparing the relative yield of PS^+^ produced (φ) to the percentage of quenched photoluminescence (%PL) determined by steady-state (Figure 5A) or time-resolved photoluminescence (Figure 5B). A concatenated linear regression of all the data points by constraining the Y-intercept at 0 provides a slope that corresponds to the cage-escape yield. R^2^ of 95% and 90% are obtained for the steady-state and time-resolved data, respectively. Cage-escape yields of 34% and 32% are obtained using the steady-state and time-resolved data, respectively.

(Equation 6) $$\Phi_{CE}=\frac{\Phi }{\%\;PL\;Quenched}$$

## Limitations

There are several limitations that may complicate such experimental protocol or render it unreliable. The first one is that the photosensitizer and the quencher must remain stable (i.e., no degradation or formation of side-products) in the dark and upon irradiation. Compounds that degrade would impact the absorbance as well as ΔAbs and hence impact the final cage-escape yields. The method that is proposed here to use individual spectrophotometric cuvettes with fresh concentration of quencher and PS allows us to potentially remediate this limitation. An additional experimental limitation is that the photosensitizer and the actinometer must absorb at the same wavelength. This is usually the case when [Ru(bpy)_3_]^2+^ is used as actinometer but may represent a limitation in some cases. Finally, the changes in absorbance between the photosensitizer in the ground state and the photosensitizer in its oxidized or reduced state must be sufficiently large to accurately determine a Δε value and the corresponding single wavelength absorption changes.

## Troubleshooting

### Dark reactivity of the photosensitizer/quencher pair

Because cage-escape experiments require relatively large concentration of both the photosensitizer and the quencher, a deterioration of the sample or the quencher solution may sometimes be observed (step 7.e). This was observed for some photosensitizers in the original report where a notable change in the absorption value at λ_exc_ was observed, indicative of ground-state oxidation.^1^

### Potential solution

The quencher can be dissolved in solvent alone (the same as for the PS solutions) but a dilution factor should be considered for the %PL Quenched values since the concentration in PS will vary with the additions. Method 2 (step 8) is preferred when using this option. If a side-reaction is occurring within the time frame of the experiment, then the results are unfortunately unreliable and should not be used.

## Resource availability

### Lead contact

Further information and requests for resources and reagents should be directed to and will be fulfilled by the lead contact. Ludovic Troian-Gautier (Ludovic.Troian@uclouvain.be)

### Materials availability

This study did not generate new unique reagents.

## Data Availability

The published article includes all datasets generated or analyzed during this study.
